# Microalgae and Cyanobacteria in the Obesity Evidence Landscape: A PRISMA-ScR Scoping Review with Mechanistic and Safety Mapping

**DOI:** 10.3390/biology15070557

**Published:** 2026-03-31

**Authors:** Quoc-Phong Huynh, Quoc-Dang Quan, Viet-The Ho, Quang-Tri Le, Huu-Cuong Nguyen, Hoang-Dung Tran

**Affiliations:** 1 Lanh Binh Thang General Hospital, 72 Street No. 5, Binh Thoi Ward, Ho Chi Minh City 72622, Vietnam; 600825001@huit.edu.vn; 2 Faculty of Biology and Environment, Ho Chi Minh City University of Industry and Trade (HUIT), 140 Le Trong Tan Street, Tay Thanh Ward, Ho Chi Minh City 72009, Vietnam; thehv@huit.edu.vn; 3 Office of Science and Technology, University of Science, VNUHCM, 227 Nguyen Van Cu Street, Cho Quan Ward, Ho Chi Minh City 72722, Vietnam; qqdang@hcmus.edu.vn; 4 Institute for Applied Research in Health Sciences and Aging, Thong Nhat Hospital, 01 Ly Thuong Kiet Street, Tan Son Nhat Ward, Ho Chi Minh City 72121, Vietnam; 5 Board of Directors, Military Hospital 175, 786 Nguyen Kiem Street, Hanh Thong Ward, Ho Chi Minh City 71423, Vietnam; tsbstri@yahoo.com; 6 Institute of Research and Application for Science and Technology Asian, 84 Huynh Van Banh Street, Cau Kieu Ward, Ho Chi Minh City 72222, Vietnam; nguyenhuucuong.vnp2@gmail.com

**Keywords:** microalgae, cyanobacteria, obesity, metabolic syndrome, PRISMA-ScR, scoping review, evidence map, mechanistic tier, safety/toxin

## Abstract

Microalgae and cyanobacteria are investigated for compounds that may relate to obesity and metabolic phenotypes, yet the evidence base remains scattered and inconsistently reported. We conducted a PRISMA-ScR scoping review of PubMed, Europe PMC, and OpenAlex and mapped eligible full-text studies to a controlled vocabulary covering intervention concepts, mechanistic pathway nodes, and obesity-related outcome domains. Of 2651 reports sought for full text, 936/2651 (35.3%) were not retrieved; therefore, evidence maps should be interpreted as conditioned on full-text accessibility. We included 836 studies and summarized study density (counts only) by population/model tier, mechanistic reporting depth, chemical class, and safety/toxin flags. The literature is concentrated in animal models (485 studies) and human studies (292), with fewer in vitro/ex vivo reports (59). Mechanistic reporting was often limited: 582 studies did not provide extractable mechanistic endpoints, whereas 254 reported biomarker or pathway “anchors”. Across intervention concepts, lipid/PUFA, carotenoids, phycobiliproteins, and polysaccharides were most common; within the anchored subset, inflammation (NF-κB), AMPK signaling, adipogenesis (PPARγ-related), and lipogenesis were the most frequently mapped nodes. Together, this evidence map clarifies where mechanistic anchoring and safety documentation are sparse and provides a clear starting point for downstream evaluation.

## Simple Summary

Microalgae and cyanobacteria contain a wide range of bioactive compounds that have been explored in obesity-related research, but the literature is dispersed across study types and often reported in ways that are hard to compare. We assembled and mapped the accessible full-text evidence to show where research density is highest, where mechanistic reporting is thin, and how often safety considerations are documented. Of 2651 reports sought for full text, 936 (35.3%) could not be retrieved, so the resulting maps reflect an evidence landscape conditioned on full-text availability. We included 836 studies and summarized them as study counts (rather than pooled effect sizes) across intervention concepts, chemical classes, mechanistic pathway nodes, and outcome domains. This scoping map is intended to support transparent prioritization and study design by making the structure of the evidence base explicit, without making efficacy or safety claims.

## 1. Introduction

In this review, “microalgae” is used as an operational literature umbrella rather than a strict phylogenetic term: the mapped corpus includes eukaryotic microalgae and cyanobacteria because both bodies of literature are frequently co-indexed and co-discussed in obesity, nutraceutical, and supplement reports. We therefore use the combined label microalgae/cyanobacteria when describing the overall evidence landscape, while distinguishing cyanobacteria explicitly where taxonomy, safety interpretation, or toxin-screening expectations differ.

Several reviews have summarized the anti-obesity potential of microalgae-derived interventions [1], but the literature remains dispersed across study types and reporting formats.

This distinction matters because names in the literature and on product labels are not fully harmonized. Records may report Arthrospira, “Spirulina”, broader cyanobacteria labels, or mixed algal products, and commercial labels do not always provide strain-level or even unambiguous genus-level resolution [2,3,4,5,6,7,8]. In the locked dataset, such names were retained traceably and normalized only to remove impossible combinations or to label genuine multi-taxa entries as Mixed/Multiple, rather than to infer exact product composition or strain identity.

Obesity is best understood as a multi-axis physiology problem rather than a single “fat accumulation” endpoint. Across human and animal studies, obesity phenotypes are commonly framed along interacting axes—lipid handling and de novo lipogenesis, chronic low-grade inflammation and insulin resistance [9], and the gut–liver axis that links barrier function, bile acids, and metabolic signaling [10]. As a result, the literature often reports panels of biochemical and tissue markers as proxies for these themes, even when outcomes are not directly comparable across studies [9,10].

Microalgae and cyanobacteria are unusual in this landscape because they act as upstream biological factories: they span broad chemistries (oils and polyunsaturated fatty acids, carotenoids, phycobiliproteins, polysaccharides, and other bioactives) and can be cultivated with controlled inputs. At the same time, the field faces a standardization challenge—taxonomic resolution, preparation formats, and reporting heterogeneity often limit what can be inferred beyond study density.

In this PRISMA-ScR scoping review, we use a controlled vocabulary and three evidence-map axes to summarize study density without implying effect size or comparative efficacy: (A) from the compound or preparation to the reported mechanism and then to phenotype; (B) from the mechanism to its marker/tissue context and then to the compound concept and taxon; and (C) from the taxon to the compound concept and then to the mechanism [11,12,13].

We provide three contributions: (i) a transparent, traceable map from intervention concepts to mechanistic pathway nodes and obesity-related outcome domains (counts-based), (ii) a marker/tissue traceability step for mechanistic reporting restricted to M1–M2 (with M3 = 0 under this fixed included-study set (*n* = 836)), and (iii) a concept-level translational workflow that links the maps to transparent downstream evaluation checklists without singling out specific strains or products.

Despite a large and growing body of work, the microalgae/cyanobacteria–obesity literature remains difficult to interpret as a coherent mechanistic field. Many studies co-report multiple intervention formats (whole biomass, oils, extracts, purified compounds) and multiple phenotype readouts, yet the chain from preparation chemistry to tissue markers is often under-specified or inconsistently labeled. Consequently, hotspots in the published record can indicate where researchers have looked most often, rather than where evidence is strongest or mechanisms are established. This interpretability gap is amplified by the fact that core obesity axes—lipid handling and de novo lipogenesis, inflammation/NF-κB-linked metaflammation, energy sensing and thermogenesis, and the gut–liver axis—are studied with heterogeneous marker panels and reporting conventions across models. Against this backdrop, a transparent scoping map with an audit trail can help separate study density from evidentiary depth and make reporting gaps visible, including the practical need for explicit toxin-exclusion/QC when cyanobacteria are involved. On that basis, this review addresses three questions: (1) What intervention concepts and chemical classes dominate the mapped literature across phenotype domains? (2) How deep is mechanistic reporting (M0–M2) and which pathway/tissue/marker panels recur in the M1–M2 subset? (3) How often are safety/toxicity endpoints reported and how does the taxonomy-based proxy for toxin risk appear within the mapped record (counts-based only) [2,3,4,5,6,7,8,14,15,16,17,18,19,20,21,22,23,24,25,26,27,28,29,30,31,32,33,34,35]?

In line with these questions, our objectives are to describe study selection and characteristics, to identify study density hotspots across these maps, and to summarize which taxa and compound classes are most frequently paired with commonly reported mechanistic panels in obesity-relevant tissues.

## 2. Materials and Methods

Design and reporting. This scoping review follows the PRISMA extension for Scoping Reviews (PRISMA-ScR) [11] and established methodological guidance for scoping reviews [12]. The flow diagram follows PRISMA 2020 templates [36], and the evidence-map framing is consistent with published evidence-mapping methods [13]. Reporting compliance is provided in Supplementary File S4. All synthesis outputs were generated from a locked dataset archived in Supplementary File S1. The technical source-of-truth for retrieval, rebuild, validation, and audit workflows was version-locked at v17 before this revision; editorial revision thereafter did not reopen searches, alter recovery outcomes, or change the included-study set (*n* = 836). No searches were rerun and no studies were added or removed after the fixed included-study set (*n* = 836).

Dataset lock and normalization. The included-study set was locked at *n* = 836. After locking, we performed only deterministic recoding and normalization steps that did not change the StudyID set and did not add or remove studies. These steps included (i) deterministic population/model tiering (human = P1; in vivo animal = P2; in vitro/ex vivo = P3); (ii) normalization of taxonomic labels to remove impossible cross-genus combinations and to label multi-taxa records as Mixed/Multiple; and (iii) the addition of descriptive eligibility/off-scope flags used only for stratified reporting. A dataset integrity summary is provided in Sheet S6_IntegrityFlags_Counts of Supplementary File S1. (Sheet S6_IntegrityFlags_Counts). Records flagged as retracted or with an expression of concern were retained under the fixed included-study set (*n* = 836) and remain included in study density counts; such records are labeled in Supplementary Table S1 and are not used to support efficacy or mechanistic claims.

Information sources and search. We searched three openly accessible bibliographic sources: PubMed, Europe PMC, and OpenAlex. Searches were executed on 14–15 December 2025 (Asia/Ho_Chi_Minh, UTC + 07) using documented platform interfaces (PubMed via NCBI E-utilities [37]; Europe PMC via REST API [38]; OpenAlex via Works API [39]). All results were exported in full via API (no sampling). No language limits or publication-type/article-type filters were applied at the search stage. Search strings and run parameters are provided in Supplementary File S1 (Sheet S1_SearchStrategy). Workflow processing and audit logging were conducted within this window; manuscript preparation and editorial revisions continued thereafter.

Eligibility criteria. Reports were eligible if they described eukaryotic microalgae, cyanobacteria, products derived from either group, or isolated compounds linked by the source report to those organisms, in relation to obesity-related phenotypes including adiposity and metabolic outcomes. Operationally, the review therefore spans both eukaryotic microalgae and cyanobacteria, but safety- and taxonomy-related interpretation distinguishes cyanobacteria explicitly when relevant. Full-text exclusion reasons were coded using a structured legend and tabulated in Supplementary File S1 (Sheet S3_FTExclusionCodes).

Selection process. Records from all sources were merged and deduplicated using a scripted pipeline in Python 3.13.5 within Google Colab (Google LLC, Mountain View, CA, USA, (Python) based on normalized identifiers (DOI/PMID) and title–year keys. After deduplication, 91,504 unique records entered title/abstract (TA) screening. TA screening was conducted in a single-reviewer, semi-automated, rule-based workflow with automated text-classification support and per-record provenance logging. A dual independent workflow was not feasible for this large corpus within available review resources; the pragmatic priority was exhaustive logged coverage and deterministic routing, not formal duplicate adjudication. TA screening retained 2651 records for full-text pursuit.

For TA-included records, full texts were pursued through a staged retrieval workflow combining automated link resolution, identifier-based source routing, open-access harvesting routes (e.g., Unpaywall [40] and Europe PMC full-text XML where available), local file materialization, and file-format/readability validation. When a payload failed to open or parse, targeted rebuild attempts were logged through alternative eligible routes in the version-locked recovery workflow. Under the v17 technical source-of-truth lock, 1715 reports were retrieved in readable form for eligibility assessment, whereas 936/2651 remained unavailable; this unavailable set included 16 residual, technically unreadable items after staged rebuild attempts, which were therefore treated as not retrieved.

Full-text screening. Retrieved full texts (*n* = 1715) were screened against prespecified eligibility criteria; 879 reports were excluded at full text with structured exclusion codes, and 836 sources of evidence were included. Screening and recording were supported by automation for routing and logging, but eligibility decisions were confirmed during reviewer full-text assessment. Independent duplicate screening and formal conflict adjudication were not performed; therefore, we did not estimate inter-rater agreement, and residual selection errors remain possible (see Limitations).

Data charting and controlled vocabulary. Data were charted into a structured extraction form and summarized into study-level tables and evidence maps. Supplementary Table S1 (Supplementary File S1, Sheet S5_TableS1_IncludedStudies) provides the included-study list and key mapped fields. Charting used a predefined controlled vocabulary for intervention form (E codes), chemical class (CC codes), pathway/mechanism nodes (PW codes), and outcome domains (O codes) (Supplementary File S1, Sheet S4_ControlledVocabulary). Multi-label fields were stored as semicolon-separated code lists; for one-code-per-study displays, we derived deterministic helper variables (Outcome_Primary_Code and Pathway_Primary_Code) to avoid double-counting in heatmaps and summary tables.

When multiple eligible outcome or pathway codes were present, the helper variable selected the first code from a prespecified ordered list defined in Supplementary File S1 (Sheet S2_PrimaryHelper). This was an operational counting rule to stabilize denominators, not a statement of biological priority, and all original multi-label fields were retained in the source table. The full-text exclusion code legend is provided in Supplementary File S1 (Sheet S3_FTExclusionCodes).

Missing or unclear reporting was handled using explicit defaults (CC0, PW0, O0, E0) as defined in the controlled vocabulary. Mapping variables for the three evidence-map axes (A–C) were generated deterministically from the charted fields and controlled-vocabulary rules.

Automation and quality assurance. Screening and data charting were supported by semi-automated, rule-based workflows implemented in Python/Google Colab to streamline deduplication, retrieval routing, deterministic recoding, helper-variable generation, and structured data charting. We applied deterministic consistency checks (e.g., code validity against the controlled vocabulary, file-state validation, and deterministic derivations used in the evidence maps) and documented reconciliation updates in the dataset notes. The version-locked scripts and rule files underpinning this workflow are referenced in the Code Availability Statement. We also conducted a post hoc, single-reviewer verification audit of 80 full texts (Supplementary File S2). Independent duplicate charting was not performed; therefore, minor residual misclassification—particularly in studies with incomplete reporting—cannot be fully excluded and should be considered when interpreting study density maps.

To strengthen traceability, we recorded page-level evidence anchors for a stratified subset of 10 audited full texts (outcome and intervention anchors; safety/toxin anchors when applicable), provided in Supplementary File S2 (Addendum).

Post hoc verification audit. Using a locally available set of 80 full texts, one reviewer compared the values assigned to the map-driving fields (chemical class, outcome domain, pathway node, population tier, mechanistic tier, taxonomy-based proxy for toxin risk, and safety/toxicity endpoint reporting flag) against the source full texts and recorded whether corrections were needed. No corrections were recorded (0/80 discrepancies for all audited fields; 100% agreement; one-sided 95% upper bound on within-sample discrepancy rate = 3.7% by Clopper–Pearson). Record-level results and summary statistics are provided in Supplementary File S2; the accompanying action log is provided in Supplementary File S3.

Tiering and mechanistic evidence definitions. Population/model was tiered as P1 (human), P2 (in vivo animal), and P3 (in vitro/ex vivo). Mechanistic depth was tiered M0–M3, where M0 indicates no qualifying mechanistic endpoints, M1 indicates associative marker evidence, M2 indicates stronger mechanistic anchoring (e.g., pathway-linked markers with tissue context), and M3 indicates causal perturbation-level evidence; in this dataset, M3 was not observed. Mechanistic_Evidence (Y/N) follows the locked definition in the codebook.

Safety/toxin handling. Cyanobacteria were flagged (Cyanobacteria_Flag) when the reported intervention taxon/label was cyanobacterial or when a mixed record contained a cyanobacterial component. Cyanobacteria_ToxinRisk_Flag was used as a conservative taxonomy-based proxy to indicate that toxin-exclusion/QC should be considered at the interpretation stage; it was applied to cyanobacteria-labelled entries (including Arthrospira/“Spirulina”-type labels and broader cyanobacteria labels) and to mixed records containing cyanobacteria, while eukaryotic microalgae-only records were coded N. This proxy does not indicate measured cyanotoxins, toxin-producing strain assignment, or confirmed toxin presence. Safety signal capture was charted using Rubric_SafetySignals (Y/N/NA). These indicators were used for mapping and as an explicit checkpoint in the downstream evaluation checklist; they do not constitute a quantitative risk assessment [2,16].

Synthesis and presentation. We summarized the evidence as counts of studies across three evidence-map axes (A–C). Counts represent study density and do not represent effect sizes or comparative efficacy. Figures include a PRISMA flow diagram, a heatmap (chemical class × outcome domain), a population-tier × mechanistic-tier bubble plot, and a safety/toxin map. Methodological choices align with established scoping-review guidance and recent updates to scoping-review conduct standards [14,15].

## 3. Results

The Section 3 should be read as an evidence map rather than an effect summary. All figures and tables report study density within the locked included set (*n* = 836) and are intended to show where the literature is concentrated, where mechanistic reporting is shallow, and where reporting conventions limit interpretability. We therefore move from broad coverage (what is studied and in which populations) to mechanistic depth (what is measured) and then to safety framing (how toxin-related considerations and safety signals are reported), keeping counts-based interpretation explicit throughout.

The results are reported as counts of included studies (study density), not as effect sizes or comparative efficacy. We first summarize the intervention landscape by chemical class and preparation concepts (evidence map A), then map outcome domains and mechanistic depth (P-tier and M-tier, including a high frequency of PW0), and finally zoom in to the marker- and tissue-anchored M1–M2 subset to describe recurring pathway–tissue–marker panels. Safety/toxin handling is presented as reporting patterns using a rule-based taxonomy-based proxy for toxin risk and a “reporting of safety/toxicity endpoints” indicator; neither should be interpreted as a quantitative risk assessment.

Here, P-tier denotes study type by population/model (P1: human; P2: in vivo animal; P3: in vitro/ex vivo), while M-tier captures mechanistic reporting depth from phenotype-only reporting (M0) to marker- and tissue-anchored (M1) and pathway-linked, marker- and tissue-anchored evidence (M2); M3 is reserved for rare causal perturbation-level designs. In this review, a “mechanistic anchor” means an explicit marker readout measured in a specified tissue and linked to a stated pathway or mechanism, enabling the consistent extraction of marker–tissue panels.

### 3.1. How to Read the Evidence Maps (Counts-Based)

The Results below are organized as a set of evidence maps. Each map reports study density (counts of mapped study records under the fixed included-study set (*n* = 836)), not effect sizes, and therefore should be read as “where the literature concentrates” rather than “what works best.”

Evidence map A translates each study into a simple chain: what was given (compound class/preparation), which mechanistic pathway node was reported (pathway), and which obesity-related phenotype domain was measured. Evidence map B adds a traceability step by linking mechanistic pathway nodes to commonly reported markers and tissues (e.g., liver, white adipose tissue, serum) and then back to compound classes and taxa. Evidence map C is taxon-centric and indicates which taxa are most often linked to which compound classes and mechanistic pathway nodes; habitat information was not reliably reported and was therefore treated as not reported.

For counting, “primary coding field” refers to the first eligible code selected from a prespecified ordered helper list when a study reports multiple codes within a map axis. This is a traceable convention to keep denominators stable; it does not imply primacy of biological importance.

Throughout, we repeat three constraints: counts are not effect estimates; mechanistic evidence is restricted to M1–M2 (M3 = 0 under this lock); and the toxin flag is a rule-based taxonomy-based proxy for toxin risk and does not indicate measured cyanotoxins or confirmed toxin presence.

### 3.2. Selection of Sources

**Figure 1:**
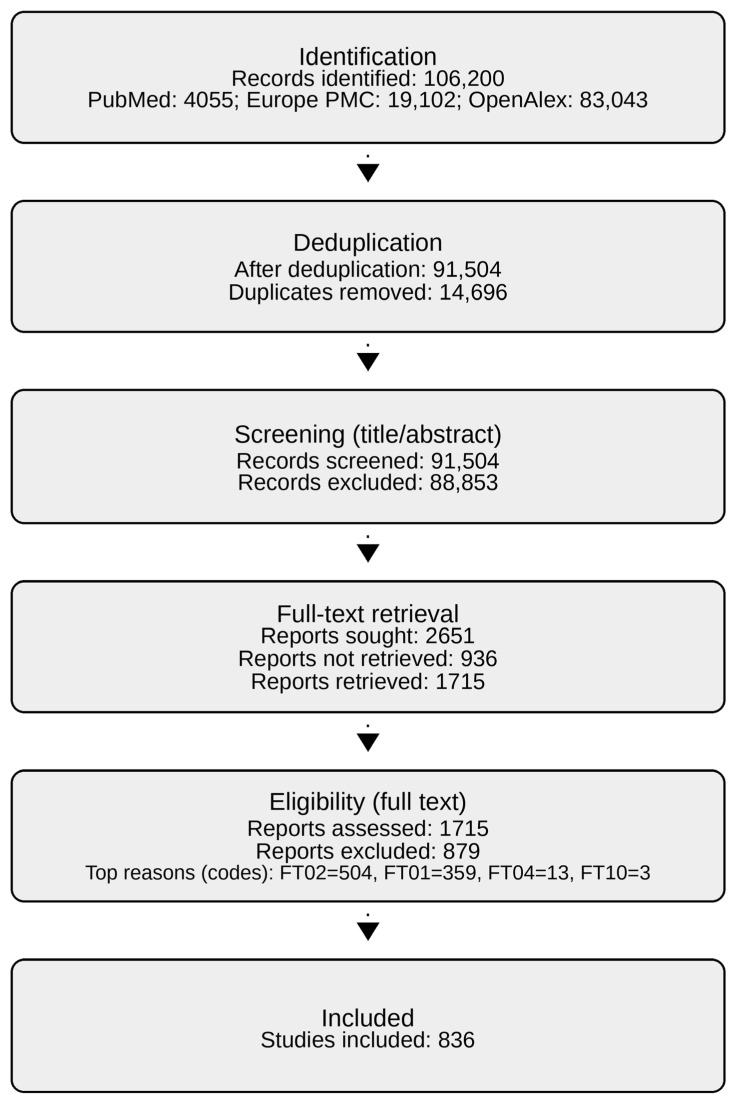
PRISMA 2020 flow diagram (scoping review). Counts reflect record/report flow only (not effect sizes). Flow counts are fixed under the fixed included study set (*n* = 836) (2651 reports sought; 936 not retrieved; 1715 retrieved; 836 included). Full-text exclusion reasons were coded using a structured legend (Supplementary File S1, Sheet S3_FTExclusionCodes).

Figure 1 summarizes selection. From 106,200 identified records, 91,504 remained after deduplication and were screened. A total of 836 studies were included in the final dataset.

### 3.3. Full-Text Retrieval and Accessibility-Conditioned Missingness

**Figure 2:**
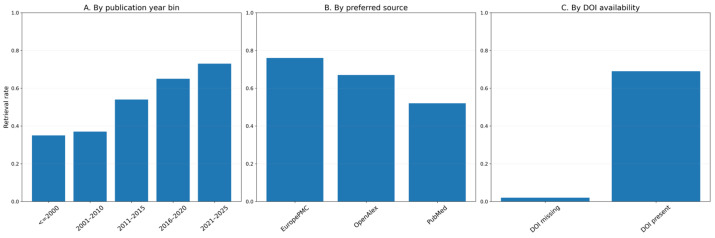
Full-text retrieval missingness patterns among reports sought (*n* = 2651): (**A**) retrieval rate by publication year bin; (**B**) retrieval rate by preferred source; (**C**) retrieval rate by DOI availability. Rates are computed as retrieved/targets within each stratum.

Of the 2651 reports sought at the end of title/abstract screening, 936/2651 (35.3%) were not retrieved under the v17 technical lock, leaving 1715 readable full texts for eligibility assessment. The unavailable set included 16 residuals, technically unreadable items after staged rebuild attempts. Retrieval rates varied by publication year bin, preferred source, and DOI availability (Figure 2A–C), underscoring that the evidence maps summarize accessible full texts rather than the full set of targets. A detailed missingness-bias summary is provided in Supplementary File S5.

### 3.4. Study Characteristics

**Figure 3:**
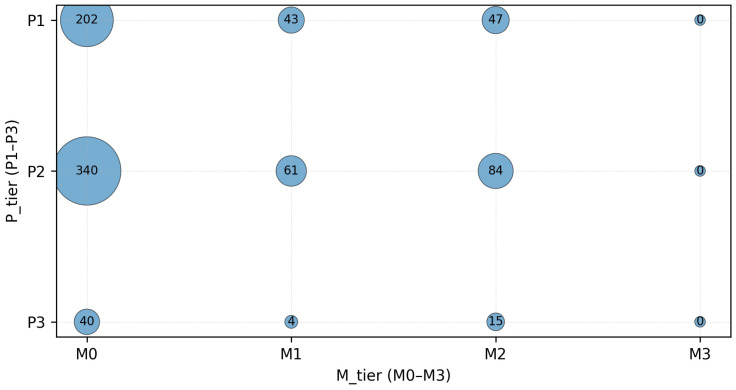
P-tier by M-tier bubble plot. Bubble size indicates number of studies (counts). P-tier: P1 is Human; P2 is Animal (in vivo); P3 is in vitro/ex vivo. M-tier: M0 indicates no qualifying mechanistic endpoints; M1 is associative marker evidence; M2 is pathway-linked marker evidence with tissue context; M3 is causal perturbation-level evidence (M3 is 0 in this dataset). Counts represent study density, not effect sizes.

Table 1 provides study-level characteristics for all 836 included studies. Most studies were in vivo animal models (P2: 485) and human studies (P1: 292); in vitro/ex vivo studies were rare (P3: 59). Mechanistic depth was predominantly M0 (582), with fewer M1 (108) and M2 (146) studies; M3 was absent. Figure 3 visualizes the joint distribution of population/model tier (P-tier) and mechanistic depth (M-tier) as a study density overview.

### 3.5. Mechanistic Patterns: Recurring Themes Across the Mapped Literature (Counts-Based)

#### 3.5.1. A Recurring Theme: Lipid Handling and De Novo Lipogenesis

**Figure 4:**
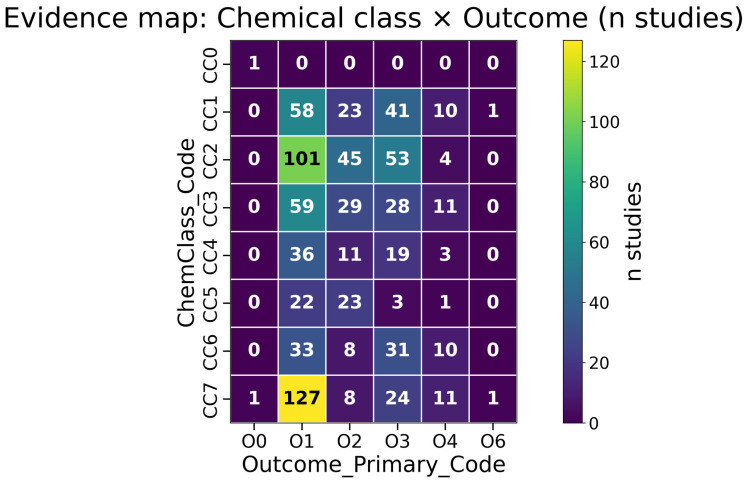
Evidence map heatmap (chemical class × primary outcome domain; primary-helper codes). Cell values are counts of included studies, not effect sizes. Code decoding: CC1 = Carotenoid; CC2 = PUFA/fatty acid; CC3 = Phycobiliprotein; CC4 = Polysaccharide; CC5 = Sterol/other lipid; CC6 = Protein/peptide; CC7 = Other/unknown; CC0 = Unknown/NR. Outcome primary (counting helper): O1 = Body weight/adiposity; O2 = Lipids; O3 = Insulin sensitivity/glucose; O4 = NAFLD/liver steatosis; O5 = Thermogenesis/energy expenditure; O6 = Inflammation; O0 = Not specified/not extracted. Note: O7 (gut barrier) and O8 (microbiome/bile acids) exist in the controlled vocabulary but did not appear as Outcome_Primary_Code in the locked dataset; gut-related outcomes may still occur in the non-primary outcome lists.

Across evidence map A, the densest clusters repeatedly connect lipid-/oil-related preparations and PUFA-rich interventions to lipid-handling outcomes and hepatic/adipose readouts. These hotspots are visible in Figure 4 and in the underlying count tables in Supplementary File S1 (Sheets S5_TableA_Full–S5_TableC_Full). When mechanistic anchoring is available (M1–M2), similar patterns recur in marker–tissue summaries (Supplementary File S1, Sheet S5_M1M2_Pathway_Tissue_Markers). Commonly reported panels in this theme include circulating lipids (e.g., TG, TC, LDL-C/HDL-C) and liver/adipose markers of lipid metabolism and storage; these should be read as reporting patterns rather than mechanistic mediation.

Taken together, the mapped record most often approaches obesity biology through lipid-handling themes, particularly via liver and white adipose tissue panels (counts-based).

#### 3.5.2. A Second Theme: Inflammation and Insulin-Resistance Signaling

A second high-density pattern links interventions—often phycobiliprotein/pigmented preparations and polysaccharide-rich extracts—to inflammation and insulin-resistance nodes. In evidence map A, these linkages cluster around inflammatory pathway codes and phenotype domains tied to glycemic control and systemic inflammation (Supplementary File S1, Sheets S5_TableA_Full–S5_TableC_Full). Where mechanistic anchoring is available (M1–M2), the most commonly reported readouts span cytokine panels and insulin sensitivity proxies measured in serum, liver, and adipose tissues (Supplementary File S1, Sheet S5_M1M2_Pathway_Tissue_Markers). This reflects what investigators tend to measure, not a mechanism hierarchy.

Likewise, inflammation and insulin-resistance signaling are frequently co-reported, but the dataset does not support causal ordering or comparative potency (counts-based; M3 = 0).

#### 3.5.3. Energy Sensing and Thermogenesis: AMPK-Centered Reporting with Adipose Tissue Anchors

A third theme highlights energy sensing and thermogenesis concepts, often expressed through AMPK-related nodes and adipose tissue remodeling readouts. In the evidence maps, these patterns appear as repeated co-occurrence between pathway codes aligned with energy sensing/mitochondrial function and phenotype domains related to energy expenditure or adiposity. They are visible in evidence map A counts and, when markers and tissues are reported, in evidence map B (Supplementary File S1, Sheets S5_TableA_Full–S5_TableC_Full and S5_M1M2_Pathway_Tissue_Markers). Commonly reported panels include AMPK-related signaling markers and thermogenesis-associated readouts in brown/white adipose tissue; these are anchors for comparability, not evidence of mediation.

AMPK/energy-sensing and thermogenesis themes also recur, yet they are typically presented as marker panels rather than tested causal chains (counts-based).

#### 3.5.4. Gut Barrier and Microbiome-Related Reporting: Present but Comparatively Sparse

Gut barrier- and microbiome-related outcomes appear in the mapped literature but at a lower study density relative to lipid and inflammation themes. When present, they tend to be reported as phenotype-domain endpoints (e.g., barrier integrity, microbiome composition proxies) with fewer consistent marker/tissue anchors under M1–M2, which limits cross-study comparability (Supplementary File S1, Sheet S5_M1M2_Pathway_Tissue_Markers). This sparsity should be interpreted as a reporting/coverage pattern under the fixed included-study set (*n* = 836), not as evidence that gut-centric mechanisms are unimportant.

Gut-axis reporting is present but less consistently anchored than liver/adipose themes within the M1–M2 subset (counts-based).

The sections below provide the traceable detail behind these patterns by reporting study selection, characteristics, and the three evidence maps (evidence map A/B/C), followed by safety/toxin proxy mapping.

Figure 4 visualizes these study density patterns as a heatmap cross-tabulating chemical class and primary outcome domain (counts only).

### 3.6. Evidence Map A: Linking Preparations to Mechanisms and Phenotypes

Evidence map A summarizes how intervention concepts (compound/preparation) map to pathway nodes and phenotype domains. Hotspots are visible in Figure 4 (chemical class × primary outcome domain), and the underlying count tables are provided in Supplementary File S1 (Sheets S5_TableA_Full–S5_TableC_Full and Top25 subsets).

### 3.7. Evidence Map B: Linking Mechanisms to Marker–Tissue Panels and Intervention Concepts

Evidence map B links mechanism nodes to specific marker–tissue contexts, compound concepts, and taxa. This axis is intended for mapping and does not imply causal mediation. The marker-anchored link summaries are reported in Table 2 and in Supplementary File S1 (Sheets S5_Table2_M1M2_LinkSummary and S5_M1M2_Pathway_Tissue_Markers).

#### 3.7.1. Mechanistic and Marker- and Tissue-Anchored Evidence (M1–M2 Only)

We restricted mechanistic enrichment to the subset with Mechanistic_Evidence = Y and M-tier ∈ {M1, M2} (*n* = 254/836 studies). Within this subset, the most frequently charted primary pathway nodes were PW3 (Inflammation/NF-κB): 115 (45.3%); PW5 (Lipogenesis): 54 (21.3%); PW1 (AMPK signaling): 38 (15.0%); PW2 (Adipogenesis/PPARγ-C/EBP): 22 (8.7%); and PW0 (Not specified/Not extracted): 19 (7.5%). Importantly, these are study density signals (counts of studies) and do not represent comparative effect sizes or efficacy, and M1/M2 endpoints do not establish causal confirmation (M3 = 0 in this dataset).

Target tissues most often linked to reported markers included White adipose tissue (WAT): 218 (85.8%), Liver: 199 (78.3%), Systemic (serum/plasma): 218 (85.8%), Brown adipose tissue (BAT): 161 (63.4%), and Intestine/gut epithelium: 148 (58.3%). Tissue–marker anchoring was commonly multi-tissue within single studies (e.g., WAT plus liver and systemic markers), reflecting mixed panels rather than single-tissue causal readouts.

Inflammation/NF-κB (PW3; *n* = 115 studies) was most commonly anchored to markers such as TNF-α (*n* = 61), IL-6 (*n* = 43), acc (*n* = 36), fas (*n* = 32), NF-κB (*n* = 27), and tnf (*n* = 24). These markers were typically measured in White adipose tissue (WAT), Liver, and systemic compartments, depending on the study design and intervention concept.

Lipogenesis (PW5; *n* = 54 studies) was most commonly anchored to markers such as acc (*n* = 48), fas (*n* = 33), lep (*n* = 15), leptin (*n* = 7), adiponectin (*n* = 5), and akt (*n* = 4). These markers were typically measured in White adipose tissue (WAT), Liver, and systemic compartments, depending on the study design and intervention concept.

AMPK signaling (PW1; *n* = 38 studies) was most commonly anchored to markers such as ampk (*n* = 28), acc (*n* = 28), fas (*n* = 26), adiponectin (*n* = 17), lep (*n* = 16), and tnf (*n* = 14). These markers were typically measured in White adipose tissue (WAT), Liver, and systemic compartments, depending on the study design and intervention concept.

Adipogenesis/PPARγ-C/EBP (PW2; *n* = 22 studies) was most commonly anchored to markers such as acc (*n* = 16), pparγ (*n* = 13), fas (*n* = 13), lep (*n* = 10), leptin (*n* = 9), and adiponectin (*n* = 7). These markers were typically measured in White adipose tissue (WAT), Liver, and systemic compartments, depending on the study design and intervention concept.

Not specified/Not extracted (PW0; *n* = 19 studies) was most commonly anchored to markers such as AKT (*n* = 9), TNF-α (*n* = 8), GLUT4/SLC2A4 (*n* = 2), and IRS-1/IRS1 (*n* = 1). These markers were typically measured in White adipose tissue (WAT), Liver, and systemic compartments, depending on the study design and intervention concept.

#### 3.7.2. Taxon to Compound to Mechanism to Marker: What Is Most Often Measured (M1–M2 Subset; Counts-Based)

Within the M1–M2 subset (mechanistic evidence present; M3 = 0 under this fixed included-study set (*n* = 836)), the charted literature repeatedly follows a recognizable measurement logic: a taxon label is paired with a compound or preparation concept, narrated through one or more mechanistic axes, and anchored—when anchoring is present—by marker panels in specific tissues. This subsection summarizes what is most often co-coded and reported as study density signals, not comparative efficacy.

A frequently observed pattern involves *Arthrospira*/“*Spirulina*” labels co-coded with phycobiliprotein concepts (e.g., phycocyanin) and most often narrated through inflammation/insulin-resistance signaling (PW3) and, in a smaller but recurrent subset, AMPK-centered reporting (PW1). When M1–M2 anchoring is available, marker panels commonly include inflammatory cytokines (e.g., TNF-α, IL-6) and energy-sensing nodes (e.g., AMPK-related readouts), typically measured in white adipose tissue (WAT), liver, and/or systemic compartments.

A second recurring pattern involves *Chlorella* labels co-coded with PUFA/oil concepts (CC2; often reported as oil/lipid preparations) and/or polysaccharide-rich preparations (CC4). These studies are often charted within the lipid-handling/lipogenesis theme (PW5) and, depending on study framing, may also co-occur with inflammation nodes (PW3). In M1–M2 records, commonly reported marker panels include lipogenesis-related nodes such as ACC, FAS, and SREBP, with tissue anchoring most frequently in liver and WAT.

Carotenoid-oriented preparations provide a third, illustrative link: *Haematococcus pluvialis* is consistently co-coded with carotenoid concepts (CC1; astaxanthin), and diatom labels (e.g., *Phaeodactylum tricornutum*) frequently co-occur with fucoxanthin concepts. When mechanistic reporting is present (M1–M2), these records are most often narrated through inflammation-adjacent or energy-sensing themes and supported by panels that overlap oxidative-stress/inflammation or AMPK-linked readouts, again with tissue context varying by design.

These patterns answer a “what is most often measured and reported” question. They do not establish causal ordering, mediation, or clinical benefit, and they should be interpreted alongside the evidence maps and the M1–M2 marker/tissue summaries as co-coding frequency under the locked dataset.

### 3.8. Evidence Map C: Linking Taxa to Compounds and Mechanisms (Habitat Not Reported)

Evidence map C maps taxa to compound concepts and pathway nodes. Habitat was not extractable in a consistent way and is therefore treated as not reported. Table C (Supplementary File S1, Sheets S5_TableC_Full and S5_TableC_Top25) summarizes the most frequent taxon–compound–mechanism links.

Across all included studies (*n* = 836), study density was highest for a small number of exact taxon labels retained in the source table, with notable heterogeneity in reporting granularity and synonym usage. The most represented exact labels were *Arthrospira platensis* (*n* = 152); *Arthrospira* sp. (*n* = 127); *Cyanobacteria* sp. (*n* = 49); *Microalgae* sp. (*n* = 47); *Chlorella vulgaris* (*n* = 47); *Phaeodactylum tricornutum* (*n* = 46); Mixed/Multiple (*n* = 44); *Chlorella* sp. (*n* = 40); *Haematococcus pluvialis* (*n* = 39); and *Chlamydomonas reinhardtii* (*n* = 25). This distribution should be interpreted as a reporting and research-focus signal rather than evidence of product prevalence. In particular, labels such as Arthrospira, “Spirulina”, broader cyanobacteria terms, and generic commercial-style descriptors often appeared at different levels of taxonomic precision across reports.

Within the taxon-to-compound-to-mechanism mapping (evidence map C), *Arthrospira*/*Spirulina*-labelled studies were most often linked to phycobiliprotein concepts (CC3; e.g., phycocyanin) and frequently lacked explicit pathway annotation (PW0). *Chlorella*-labelled studies were more frequently linked to PUFA/oil concepts (CC2/E5) and polysaccharide concepts (CC4), with more frequent mapping to lipogenesis (PW5) and inflammation (PW3) nodes. *Haematococcus pluvialis* was consistently associated with carotenoid concepts (CC1; astaxanthin) in the included literature. Strain identifiers, habitat provenance, and batch characterization were frequently not reported, precluding inference about specific strains or environmental sources.

### 3.9. Safety/Toxin Mapping

**Figure 5:**
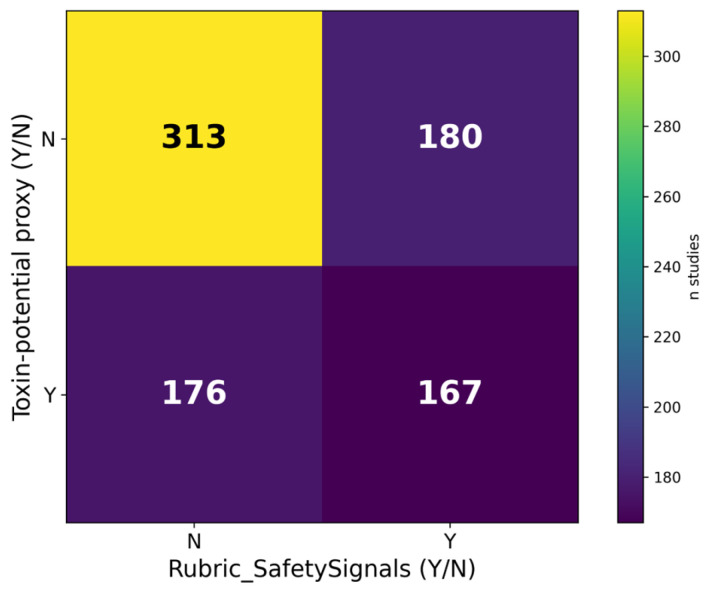
Safety/toxin map: cyanobacteria taxonomy-based proxy for toxin risk × reporting of safety/toxicity endpoints (counts of studies). “Toxin-risk” is a taxonomy-based proxy for toxin risk (cyanotoxins were not measured and presence is not implied). “Reporting of safety/toxicity endpoints” indicates whether a study reported any safety/toxicity endpoints (Y) versus not captured/not reported (N); N should not be interpreted as evidence of safety.

The reporting of safety/toxicity endpoints and cyanobacteria toxin-risk flags was mapped at the study level. Figure 5 summarizes counts by toxin-risk flag and whether safety signals were captured. The proxy flag was triggered by cyanobacteria-labelled records or mixed records containing cyanobacteria and should be read as a conservative QC cue rather than a measured toxin variable. These indicators highlight reporting gaps; counts are not estimates of exposure or risk, and they underscore the need for toxin-exclusion/QC prior to any candidate claims, especially for cyanobacteria entries, where transparent toxin-exclusion/QC reporting would be expected in translational evaluation; the flag used here is taxonomy-based and does not imply toxin presence.

## 4. Discussion

Across the dataset, the maps show broad coverage but limited mechanistic interpretability. Hotspots largely reflect where studies cluster and how materials are labeled; the predominance of M0 and the absence of M3 (M3 = 0) indicate that comparable causal, target-engagement chains are rarely established. The main bottleneck is therefore standardization—how preparations are characterized, how pathways are annotated, and how tissue–marker anchors are reported—alongside uneven safety/toxicity documentation and the need for explicit toxin-exclusion/QC when cyanobacteria are involved. In this setting, mapping is most useful for distinguishing volume from interpretability and for specifying a minimum reporting set before stronger mechanistic or translational claims are attempted [15,17].

Versioning note for interpretation. The P-tier distribution reported here reflects deterministic integrity recoding and taxon normalization applied after the fixed included-study set (*n* = 836) (human = P1; animal = P2; in vitro/ex vivo = P3). These changes improve the internal consistency of study density mapping but do not change the included-study set (*n* = 836) and should not be interpreted as a biological shift in the underlying literature.

Illustrative pattern 1 (phycobiliprotein-oriented theme). Across studies co-coded as phycobiliprotein-related concepts (CC3) in animal and human tiers, a common reporting pattern links adiposity or lipid outcomes (O1–O2 helper codes) to inflammation-leaning nodes (PW3 helper) with recurring cytokine panels. These studies frequently report WAT and liver as anchor tissues, with marker tokens such as TNF-α, IL-6, and NF-κB family readouts appearing as a combined panel rather than a single pathway claim. Notably, the PW3 label is a counting helper derived from extracted code lists; marker tokens can be shared across themes and should not be read as pathway-specific proof. Within the M1–M2 subset, this theme appears as a dense reporting pattern, but it does not establish comparative efficacy or causality. Representative examples in the locked included set are summarized in Table 2 and are traceable in Supplementary Table S1.

Illustrative pattern 2 (algal oil/PUFA theme). For PUFA/fatty-acid concepts (CC2), the mapped record more often anchors interpretation in lipid handling and de novo lipogenesis (PW5 helper) alongside serum lipid outcomes (O1–O2 helper codes). Across tiers, liver and systemic/serum panels are commonly charted, with lipogenic marker tokens such as ACC, FAS, SREBP, and related enzymes frequently extracted where mechanistic endpoints are present. Again, these marker tokens are reported verbatim and aggregated for mapping; their presence reflects reporting density and does not imply a validated causal chain. Representative included examples are summarized in Table 2 and are traceable in Supplementary Table S1.

Illustrative pattern 3 (carotenoid theme). Carotenoid-oriented concepts (CC1), including fucoxanthin-labelled interventions, frequently co-occur with AMPK-related signaling labels (PW1 helper) and mixed phenotype domains spanning adiposity, lipids, and insulin sensitivity. In the M1–M2 subset, studies often chart AMPK/PGC-1α/PPAR-family tokens across liver, WAT, and muscle, reflecting a recurring energy-sensing and lipid-handling framing in the literature. As elsewhere, PW1 is a primary-helper mechanism label used to avoid double counting in heatmap summaries; it should not be interpreted as proof of a singular mechanism. Representative included examples are summarized in Table 2 and are traceable in Supplementary Table S1.

### 4.1. Discussion Overview and Synthesis Anchors

This PRISMA-ScR scoping review mapped a large and diverse body of literature (836 included studies) on microalgae/cyanobacteria-derived interventions in obesity-related contexts. The evidence base is concentrated in animal models and, to a lesser extent, human studies, with relatively few in vitro/ex vivo studies. However, across the dataset, mechanistic depth is limited: most studies lack qualifying mechanistic endpoints (M0), and none reached the strongest mechanistic tier (M3). Accordingly, this synthesis should be interpreted as a landscape map rather than confirmation of mechanism or clinical efficacy.

Hotspots in evidence map A indicate recurring combinations of intervention concepts and phenotype domains, particularly around lipid/oil preparations and phycobiliprotein-related concepts. At the same time, the frequent assignment to broad pathway nodes (PW0) suggests that pathway annotation is often absent or not standardized, limiting cross-study integration. Future primary research would benefit from the harmonized reporting of chemical characterization, dosing, and mechanistic endpoints anchored to tissue–marker contexts.

Key mechanistic answers (study density; M1–M2 subset). (1) Which taxa and compounds are most often linked to mechanistic readouts: links were most frequently reported for Arthrospira/Spirulina-labelled interventions, including phycobiliproteins (e.g., phycocyanin) and lipid/oil concepts, followed by Chlorella-labelled interventions and carotenoid-rich taxa (e.g., Haematococcus pluvialis for astaxanthin). (2) Which pathways are most frequently implicated: PW3 (inflammation/NF-κB), PW5 (lipogenesis), PW1 (AMPK), and PW2 (adipogenesis/PPARγ-C/EBP) dominated the M1–M2 subset. (3) Which tissues/markers are most frequently used: WAT and liver (plus systemic panels) dominated, with frequent reporting of TNF-α and IL-6 (inflammation), ACC/FAS and SREBP (lipogenesis), AMPK (energy sensing), and PPARγ/C/EBP-related markers (adipogenesis). These summaries describe what is most often measured (Table 2 and Supplementary File S1, Sheet S5_M1M2_Pathway_Tissue_Markers) and should not be interpreted as comparative efficacy.

### 4.2. What We Can and Cannot Infer from a Counts-Based Scoping Map

What is robust under this fixed included-study set (*n* = 836) is the structure of study density: which compound/preparation concepts most often co-occur with which mechanism nodes and phenotype domains, and which marker/tissue panels are most frequently used when mechanistic reporting is present (M1–M2). These patterns help readers navigate the literature and identify where replication and standardization efforts may be most informative. At a concept level, the mapped record most often frames phycobiliprotein-oriented interventions against inflammation/insulin-resistance panels, lipid/PUFA-rich preparations against liver/adipose lipid-handling panels, and carotenoid-rich interventions against energy-sensing/AMPK-adjacent panels; however, these are pharmacology-oriented reporting clusters, not effect estimates.

What is not inferable here is comparative efficacy, causal mechanism, or strain-level candidacy. This review does not compute effect sizes or head-to-head comparisons, and mechanistic depth is limited to M1–M2 with M3 = 0; therefore, any language implying mediation, superiority, or translation to products/clinical practice would exceed the claim ceiling. A small number of included records carry a Retraction_EoC_Flag and are kept only as part of the locked evidence landscape; they should be interpreted with caution and do not support efficacy conclusions.

### 4.3. Why Mechanism Reporting Is the Bottleneck (And Why PW0 Dominates)

A prominent feature of the evidence maps is the large “PW0” region—records where mechanisms were not reported at a level that could be charted into the predefined pathway vocabulary. This should be read primarily as a reporting and extraction constraint (what authors report, what full texts are available, and how consistently outcomes are described), not as evidence of biological absence.

A practical implication is that future studies may improve cross-study interpretability by reporting a minimum, interoperable set: taxonomic identification with transparent markers, a clearly defined preparation format and dose basis, a small set of tissue-anchored mechanistic panels aligned to the major themes (liver/adipose/serum; lipid handling; inflammation/insulin signaling; energy sensing/thermogenesis), and explicit safety/toxin handling when cyanobacteria are involved.

Linking the maps to next-step checklists. The evidence maps can support concept-level “what to measure next” and “what to test next” checklists for downstream evaluation, but they are not intended to single out strains or products, nor to support clinical recommendations.

### 4.4. From Evidence Maps to a Concept-Level Evaluation Checklist

Table 3 offers a conceptual overview linking recurring preparation archetypes (Modules A–E) to mechanistic axes that are commonly charted in this literature. It is provided solely as a reader aid (conceptual, not data-derived) and does not imply causality, efficacy, or any ranking of taxa or compounds.

Together, Table 3 and Table 4 provide a concept-level roadmap that connects the scoping evidence maps to a transparent downstream evaluation checklist, without functioning as a selection tool.

First, before any downstream interpretation, a given material/source can be documented with (1) taxonomic identification with appropriate barcodes (e.g., 16S/18S/ITS/23S as applicable) and traceable culture provenance; (2) basic growth and manufacturability metrics (growth rate, biomass productivity, culture stability, tolerance ranges for salinity/light/temperature); (3) chemical profiling (an LC–MS/MS fingerprint plus targeted quantification for 1–3 compound classes selected based on the intended evaluation hypothesis; the maps indicate what is commonly reported, not what is present in a given material/source); (4) optional genomic signals where relevant (e.g., pathway genes or biosynthetic loci supporting the measured chemistry); (5) screening bioassays aligned to a mechanism node (e.g., adipogenesis proxy, inflammation/NF-κB proxy, AMPK proxy) alongside cytotoxicity, explicitly treated as screening rather than proof; and (6) for cyanobacteria, explicit toxin-exclusion/QC reporting that distinguishes proxy flags from measured toxins.

Second, several constraints help keep concept-level evaluation interpretable. When cyanobacteria are involved, reporting should clearly distinguish taxonomy-based proxy for toxin risks from measured toxins and include toxin-exclusion/QC information when available; the absence of reporting is not evidence of absence. Cytotoxicity information can be treated as a concept-level screen (with predefined thresholds appropriate for the assay and intended exposure context). Finally, identity and contamination control should be explicitly documented (barcoding confirmation, culture purity checks, batch traceability), because misidentification, mixed products, or contamination can invalidate mechanistic attribution and can break the link between a marketed label and the taxon/preparation actually tested [2,3,4,5,6,7,8].

Third, Table 4 (MCDA/EtD, a multi-criteria evidence-to-decision (EtD) checklist) is intended as a transparency aid for research planning; it is not a clinical recommendation, regulatory instrument, or risk-of-bias tool. If a criterion is not reported, it should be recorded as NA/not scorable, and missing data should not be backfilled or inferred from related fields. The checklist makes the next measurements explicit (what to measure next and which criteria should be met) before escalation to more resource-intensive studies.

Fourth, this review does not select specific strains or products. Strain collections and repositories are intentionally out of scope for this PRISMA-ScR because selection requires empirical profiling (chemistry, bioassays, and QC) that cannot be inferred from study density maps. To avoid overclaim and to preserve QA/QC traceability, any strain screening and selection should be reported as separate original research with prespecified assays, thresholds, and reporting standards.

### 4.5. Key Messages (Counts-Based; No Efficacy Claims)

- Across the mapped literature, study density concentrates in a small number of mechanistic axes (lipid handling; inflammation/insulin signaling; energy sensing/thermogenesis), but counts do not indicate effect size.
- Compound classes are most often reported as preparation concepts (e.g., PUFA-rich oils, carotenoids, phycobiliproteins, polysaccharides) rather than standardized molecules, which limits comparability.
- Mechanistic anchoring relies on recurring tissue/marker panels (liver, white/brown adipose tissue, serum), yet mechanistic depth remains limited to M1–M2 with M3 = 0 under this lock.
- The high frequency of PW0 should be interpreted as a reporting/extraction bottleneck; improving minimum reporting standards is likely higher leverage than expanding outcome lists.
- Any translation to strain discovery must remain concept-level and traceable: no strain selection, no product/clinical recommendations, and explicit toxin-handling constraints for cyanobacteria.

Limitations. A key limitation is incomplete full-text coverage under the fixed included-study set (*n* = 836): of 2651 reports sought, 936/2651 (35.3%) were not retrieved under the v17 technical source-of-truth, and this inaccessible set included 16 residual, technically unreadable items after staged recovery/rebuild attempts. This may systematically under-represent parts of the literature (e.g., older, paywalled, poorly indexed, or identifier-incomplete reports) and can shift apparent study density hotspots toward more recent, DOI-complete, and open-access-friendly literatures. Accordingly, the mapped landscape should be interpreted as conditioned on full-text accessibility rather than as a census of all potentially relevant reports. Second, title/abstract screening, full-text eligibility assessment, and data charting were performed in a single-reviewer workflow supported by automation, deterministic rules, and logged provenance. This pragmatic design improved feasibility for a very large corpus, but it departs from optimal dual independent PRISMA-ScR practice and leaves a residual risk of selection and extraction bias. To partially bound this risk, we conducted a post hoc, single-reviewer verification audit of 80 locally available included reports; no corrections were recorded for the audited map-driving fields (Supplementary File S2), corresponding to a one-sided 95% upper bound of 3.7% on the within-sample discrepancy rate. Third, mechanism-node mapping was frequently under-specified: the primary pathway helper PW0 (“not specified/not extracted”) accounts for 551/836 studies (65.9%), reducing the interpretability of mechanistic clustering and increasing sensitivity to reporting omissions. Finally, the cyanobacteria toxin indicator should be interpreted strictly as a taxonomy-based proxy for toxin risk rather than measured cyanotoxin exposure; the safety/toxin map is a structured caution signal and not a quantitative risk assessment. A small subset of records carried audit labels indicating unclear primary-study status or topical scope; these records were retained to preserve traceability and may slightly dilute the topical specificity of counts. Together, these constraints reinforce that this scoping review supports evidence mapping and research planning rather than efficacy or safety claims.

## 5. Conclusions

Within the locked evidence landscape, three concept-level clusters dominate obesity-related reporting: phycobiliprotein-oriented interventions are most often charted against inflammation/insulin-resistance panels; lipid/PUFA-rich preparations are most often charted against lipid-handling and liver/adipose marker panels; and carotenoid-oriented preparations are most often charted against energy-sensing/AMPK-adjacent panels. These are study density patterns, not comparative pharmacological effect estimates, and they do not establish causal mechanism or product-level efficacy.

The practical value of this PRISMA-ScR is therefore to clarify what future studies should report more transparently before stronger claims are made: taxonomic identity, preparation chemistry, dose basis, tissue-anchored mechanistic panels, and explicit safety/toxin exclusion and QC, especially when cyanobacteria or cyanobacteria-containing products are involved. Because the map is conditioned on accessible full texts and shaped by heterogeneous labeling/reporting, it should be used as a traceable navigation and research-planning tool rather than as a basis for clinical recommendations or safety conclusions.

## Figures and Tables

**Table 1:** Study characteristics (excerpt; full included studies table provided as Supplementary Table S1 in Supplementary File S1, Sheet S5_TableS1_IncludedStudies).

StudyID	Year	Study_Model	P_Tier	Intervention_Form	Taxon_Canon	ChemClass_Code	Bioactive_Compound	Outcome_Primary_Code	Mechanistic_Evidence	M_Tier
S1-0223	2000	Human study	P1	E5	*Arthrospira* sp.	CC2	NR	O1	N	M0
S1-0234	2006	Human study	P1	E5	*Crypthecodinium cohnii*	CC2	DHA	O1	N	M0
S1-0235	2006	Human study	P1	E5	*Microalgae* sp.	CC2	DHA	O1	N	M0
S1-0002	2008	Human study	P1	E7	*Arthrospira platensis*	CC7	phycocyanin; epa; spirulina; arthrospira	O1	Y	M1
S1-0229	2008	Human study	P1	E5	*Microalgae* sp.	CC2	DHA	O2	N	M0
S1-0542	2008	Human study	P1	E7	*Arthrospira platensis*	CC3	Phycocyanin	O1	N	M0
S1-0233	2009	Human study	P1	E6	*Arthrospira* sp.	CC4	NR	O2	Y	M2
S1-0006	2011	Human study	P1	E6	*Arthrospira* sp.	CC7	polysaccharide; epa; spirulina	O1	Y	M2
S1-0771	2011	Human study	P1	E8	*Chlamydomonas* sp.	CC6	NR	O1	N	M0
S1-0779	2011	Human study	P1	E1	*Chlorella* sp.	CC6	NR	O1	N	M0
S1-0835	2011	Human study	P1	E6	*Chlorella vulgaris*	CC4	NR	O1	N	M0
S1-0760	2012	Human study	P1	E1	*Arthrospira platensis*	CC6	NR	O1	N	M0
S1-0824	2012	Human study	P1	E7	Mixed/Multiple	CC1	Astaxanthin	O2	N	M0
S1-0839	2012	Human study	P1	E6	*Chlorella vulgaris*	CC2	EPA	O2	N	M0
S1-0276	2013	Human study	P1	E5	*Chlorella* pyrenoidosa	CC2	NR	O1	N	M0
S1-0393	2013	In vitro/ex vivo	P3	E5	*Chlamydomonas* sp.	CC2	NR	O2	N	M0
S1-0547	2013	Human study	P1	E5	*Chlorella* sp.	CC2	EPA	O1	N	M0
S1-0565	2013	Human study	P1	E4	*Haematococcus* sp.	CC1	Astaxanthin	O1	N	M0
S1-0011	2014	Human study	P1	E5	NR	CC2	epa	O1	Y	M1
S1-0013	2014	Human study	P1	E7	*Arthrospira platensis*	CC7	phycocyanin; c-phycocyanin; dha; epa; spirulina; arthrospira	O1	Y	M2

**Table 2:** Taxon–compound–pathway–marker link summary (study density; M1–M2 subset).

Taxon_Canon	Compound Concept	Pathway Node	Tissue	Marker (Verbatim)	n_Studies	n_P1	n_P2	n_P3	M1/M2
*Arthrospira platensis*	Phycocyanin	PW0—Not specified/Not extracted	White adipose tissue (WAT); Liver; Brain/hypothalamus; Systemic (serum/plasma)	TNF-α	2	0	2	0	1/1
*Arthrospira platensis*	Other/unknown|Phycobiliprotein	PW1—AMPK signaling	Brown adipose tissue (BAT); Liver; Skeletal muscle; Intestine/gut epithelium; Immune cells/macrophages; Systemic (serum/plasma)	Fas; acc; ampk; akt; adiponectin; tnf-α; tnf; nf-κb	1	1	0	0	0/1
*Arthrospira platensis*	Other/unknown|Phycobiliprotein	PW3—Inflammation/NF-κB	White adipose tissue (WAT); Brown adipose tissue (BAT); Liver; Skeletal muscle; Intestine/gut epithelium; Brain/hypothalamus; Immune cells/macrophages; Systemic (serum/plasma)	fas; acc; adiponectin; leptin; lep; tnf	1	1	0	0	0/1
*Arthrospira platensis*	Other/unknown|Phycobiliprotein	PW3—Inflammation/NF-κB	White adipose tissue (WAT); Liver; Intestine/gut epithelium; Brain/hypothalamus; Immune cells/macrophages; Systemic (serum/plasma)	fas; acc; adiponectin; tnf-α; tnf; il-6	1	1	0	0	0/1
*Arthrospira platensis*	Other/unknown|Polysaccharide	PW5—Lipogenesis	White adipose tissue (WAT); Brown adipose tissue (BAT); Liver; Intestine/gut epithelium; Systemic (serum/plasma)	fas; acc; ucp1	1	1	0	0	0/1
*Arthrospira platensis*	PUFA/fatty acid|Oil/lipid	PW2—Adipogenesis/PPARγ-C/EBP	White adipose tissue (WAT); Brown adipose tissue (BAT); Liver; Skeletal muscle; Systemic (serum/plasma)	PPARγ/PPARG	1	1	0	0	0/1
*Arthrospira platensis*	Phycocyanin	PW0—Not specified/Not extracted	White adipose tissue (WAT); Brown adipose tissue (BAT); Liver; Skeletal muscle; Intestine/gut epithelium; Systemic (serum/plasma)	AKT	1	1	0	0	0/1
*Arthrospira platensis*	Phycocyanin	PW2—Adipogenesis/PPARγ-C/EBP	White adipose tissue (WAT); Liver; Skeletal muscle; Intestine/gut epithelium; Brain/hypothalamus; Immune cells/macrophages; Systemic (serum/plasma)	PPARγ/PPARG; TNF-α; IL-6; AKT	1	1	0	0	0/1
*Arthrospira platensis*	Phycocyanin	PW3—Inflammation/NF-κB	White adipose tissue (WAT); Brown adipose tissue (BAT); Intestine/gut epithelium; Brain/hypothalamus; Immune cells/macrophages; Systemic (serum/plasma)	NF-κB; TNF-α	1	1	0	0	0/1
*Arthrospira* sp.	Other/unknown|Phycobiliprotein	PW1—AMPK signaling	White adipose tissue (WAT); Brown adipose tissue (BAT); Liver; Intestine/gut epithelium; Immune cells/macrophages; Systemic (serum/plasma)	fas; acc; ampk; adiponectin; leptin; lep; tnf-α; tnf	1	1	0	0	0/1
*Arthrospira* sp.	Other/unknown|Phycobiliprotein	PW3—Inflammation/NF-κB	Liver; Immune cells/macrophages; Systemic (serum/plasma)	fas; acc; tnf-α; tnf	1	1	0	0	0/1
*Arthrospira* sp.	Other/unknown|Phycobiliprotein	PW3—Inflammation/NF-κB	White adipose tissue (WAT); Brown adipose tissue (BAT); Liver; Intestine/gut epithelium; Immune cells/macrophages; Systemic (serum/plasma)	fas; acc; leptin; lep; il-6; tlr4	1	1	0	0	0/1
*Arthrospira* sp.	Other/unknown|Phycobiliprotein	PW5—Lipogenesis	White adipose tissue (WAT); Liver; Skeletal muscle; Intestine/gut epithelium; Systemic (serum/plasma)	fas; acc; lep	1	1	0	0	0/1
*Arthrospira* sp.	Other/unknown|Polysaccharide	PW3—Inflammation/NF-κB	Liver; Skeletal muscle; Intestine/gut epithelium; Immune cells/macrophages; Systemic (serum/plasma)	fas; acc; adiponectin; il-6	1	1	0	0	0/1
*Arthrospira* sp.	Other/unknown|Polysaccharide	PW3—Inflammation/NF-κB	White adipose tissue (WAT); Brown adipose tissue (BAT); Liver; Intestine/gut epithelium; Immune cells/macrophages; Systemic (serum/plasma)	fas; acc; adiponectin; lep; tnf-α; tnf; il-6; il6; mcp-1	1	1	0	0	0/1
*Arthrospira* sp.	Phycocyanin	PW0—Not specified/Not extracted	Liver; Skeletal muscle; Intestine/gut epithelium; Systemic (serum/plasma)	GLUT4/SLC2A4	1	1	0	0	0/1
*Arthrospira* sp.	Phycocyanin	PW3—Inflammation/NF-κB	White adipose tissue (WAT); Brown adipose tissue (BAT); Liver; Intestine/gut epithelium; Immune cells/macrophages; Systemic (serum/plasma)	TNF-α; IL-6; TGR5/GPBAR1	1	1	0	0	0/1
*Arthrospira* sp.	Phycocyanin	PW3—Inflammation/NF-κB	White adipose tissue (WAT); Liver; Immune cells/macrophages	TNF-α	1	1	0	0	0/1
*Arthrospira* sp.	Phycocyanin	PW3—Inflammation/NF-κB	White adipose tissue (WAT); Liver; Intestine/gut epithelium; Immune cells/macrophages	NF-κB; TNF-α; IL-6	1	1	0	0	0/1
*Arthrospira* sp.	Polysaccharide|Polysaccharide	PW3—Inflammation/NF-κB	White adipose tissue (WAT); Intestine/gut epithelium; Immune cells/macrophages; Systemic (serum/plasma)	NF-κB	1	1	0	0	0/1
*Chlorella* sp.	PUFA/fatty acid|Oil/lipid	PW4—Bile acids/FXR-TGR5	White adipose tissue (WAT); Liver; Skeletal muscle	SREBP	1	1	0	0	0/1
*Chlorella* sp.	Polysaccharide|Polysaccharide	PW3—Inflammation/NF-κB	White adipose tissue (WAT); Brown adipose tissue (BAT); Liver; Skeletal muscle; Immune cells/macrophages; Systemic (serum/plasma)	NF-κB; IL-6	1	1	0	0	0/1
*Chlorella vulgaris*	Other/unknown|Phycobiliprotein	PW3—Inflammation/NF-κB	White adipose tissue (WAT); Brown adipose tissue (BAT); Liver; Intestine/gut epithelium; Immune cells/macrophages; Systemic (serum/plasma)	fas; acc; akt; leptin; lep; tnf-alpha; tnf	1	1	0	0	0/1
*Chlorella vulgaris*	Other/unknown|Pure compound	PW3—Inflammation/NF-κB	White adipose tissue (WAT); Brown adipose tissue (BAT); Liver; Immune cells/macrophages; Systemic (serum/plasma)	fas; acc; adiponectin; leptin; lep; nf-κb	1	1	0	0	0/1
Cyanobacteria sp.	PUFA/fatty acid|Oil/lipid	PW3—Inflammation/NF-κB	White adipose tissue (WAT); Liver; Intestine/gut epithelium; Systemic (serum/plasma)	TNF-α	1	1	0	0	0/1

Display rule: Table 2 lists 25 link rows to keep the main text readable. Rows are ordered by (i) n_studies; then (ii) n_M2, (iii) n_M1, and (iv) P-tier coverage (n_P1, n_P2, n_P3); and then (v) alphabetical taxon label. Counts represent the number of studies and do not indicate comparative efficacy. The full link summary table is provided in Supplementary File S1 (Sheet S5_Table2_M1M2_LinkSummary).

**Table 3:** Concept schematic linking preparation modules (A–E) to recurring mechanistic axes and illustrative marker–tissue panels (conceptual schematic; not a data-derived analysis).

Module	Lipogenesis	Glucose Handling	Inflammation/Immune Response/Polarization	Expenditure/Thermogenesis	Gut Barrier/Microbiota-Linked Host Response	Oxidative Stress/Redox
A. Polysaccharides/EPS-rich fractions	L, W, M	G, W	—	—	G	G, L
B. Protein/peptide hydrolysates	—	W	W	—	—	—
C. Redox-active fractions (e.g., phenolics, phycobilins)	W	L, W	W	—	—	L, W
D. Fucoxanthin/carotenoid-rich fractions	W, M	—	W, BAT	G	—	L
E. Polar lipid/glycolipid fractions	L, W	L, W	—	—	G (opt.)	L

Legend: L = liver; W = white adipose tissue (visceral preferred); BAT = brown adipose tissue; G = gut; M = skeletal muscle. Note: Example panels illustrate example interrogation targets for subsequent studies; they are not evidence of demonstrated mechanisms.

**Table 4:** Mechanistic themes (evidence-map domains) and their closest clinical translation proxy endpoints (conceptual).

Mechanistic Theme (Evidence-Map Domain)	Closest Clinical Translation Proxy Endpoint Group (Conceptual)
Hepatic lipid handling and lipid storage (ACC/FAS/SREBP1c; hepatic triglycerides; hepatic lipid content)	Liver triglycerides/NAFLD–NASH proxies; plasma lipid panel (TC/TG/HDL/LDL)
Adipose tissue biology and fat-mass remodeling (PPARγ, C/EBPα; adipocyte size; white adipose tissue—WAT)	Body composition (fat mass); adipocyte size (if available); adipokines (leptin/adiponectin)
Inflammation and immune polarization (NF-κB, TNF-α/IL-6; macrophage infiltration)	Inflammation proxy group (e.g., hsCRP); cytokine panel; immune cell infiltration markers
Glucose homeostasis and insulin signaling (HOMA-IR; glucose uptake/AKT signaling)	Glucose handling/insulin sensitivity proxies; fasting glucose/insulin; oral glucose tolerance-type measures (as available)
Gut barrier and endotoxemia (LPS; tight junction markers; LPS/LBP; microbiota signals)	Gut–liver axis proxy group; leaky-gut markers; short-chain fatty acids (SCFAs); gut-derived metabolite proxies (as available)
Safety/toxin handling (cyanobacteria-linked) (toxin exposure signals; cellular toxicity)	Toxicity/QC reporting requirement (conceptual): report toxin-related QC explicitly; the absence of evidence is not evidence of absence

## Data Availability

The locked dataset and derived tables supporting the findings of this scoping review are provided in Supplementary File S1 (Supplementary_File_S1_Supplement_Version2.xlsx). Version-locked scripts, rule files, and reproducibility notes supporting deduplication, retrieval routing, deterministic recoding, helper-variable generation, consistency checks, and figure assembly are available as part of the shareable reproducibility package at https://doi.org/10.5281/zenodo.19222366. Source full-text files are not publicly redistributed.

## Supplementary Materials

The following supporting information can be downloaded at https://www.mdpi.com/article/10.3390/biology15070557/s1, Supplementary File S1: Supplementary_File_S1_Supplement_Version2.xlsx (Sheets S1_SearchStrategy, PRISMA_Flow, S2_PrimaryHelper, S3_FTExclusionCodes, S4_ControlledVocabulary, S5_TableS1_IncludedStudies, and derived summary tables); Supplementary File S2: Supplementary_File_S2_ValidationAudit_Version1.xlsx; Supplementary File S3: Supplementary_File_S3_Audit80_ActionLog_Version1.csv; Supplementary File S4: Supplementary_File_S4_PRISMA-ScR_Checklist_Version2.docx; Supplementary File S5: Supplementary_File_S5_MissingnessBias_Report_Version2.xlsx.

## Abbreviations

The following abbreviations are used in this manuscript:

ACC	acetyl-CoA carboxylase
AKT	protein kinase B
AMPK	AMP-activated protein kinase
API	application programming interface
BAT	brown adipose tissue
CC	chemical class code (controlled vocabulary)
C/EBP	CCAAT/enhancer-binding protein
DOI	digital object identifier
E (codes)	intervention-form codes (controlled vocabulary)
EtD	evidence-to-decision
FAS	fatty acid synthase
FXR	farnesoid X receptor
GLUT4/SLC2A4	glucose transporter type 4
HDL	high-density lipoprotein cholesterol
HOMA-IR	homeostatic model assessment of insulin resistance
hsCRP	high-sensitivity C-reactive protein
IL-6	interleukin-6
IRS-1/IRS1	insulin receptor substrate-1
LBP	lipopolysaccharide-binding protein
LC–MS/MS	liquid chromatography–tandem mass spectrometry
LDL	low-density lipoprotein cholesterol
MCDA	multi-criteria decision analysis
M-tier (M0–M3)	mechanistic reporting depth tiers (M0 phenotype-only; M1 marker evidence; M2 pathway-linked marker evidence with tissue context; M3 causal perturbation-level evidence; M3 = 0 in this dataset)
NAFLD	non-alcoholic fatty liver disease
NASH	non-alcoholic steatohepatitis
NCBI	National Center for Biotechnology Information
NF-κB	nuclear factor kappa B
O (codes)	outcome-domain codes (controlled vocabulary)
OA	open-access
P-tier (P1–P3)	population/model tiers (P1 human; P2 in vivo animal; P3 in vitro/ex vivo)
PMID	PubMed identifier
PPARγ	peroxisome proliferator-activated receptor gamma
PRISMA	Preferred Reporting Items for Systematic Reviews and Meta-Analyses
PRISMA-ScR	PRISMA Extension for Scoping Reviews
PUFA	polyunsaturated fatty acids
PW	pathway/mechanism node code (controlled vocabulary)
QC	quality control
SCFAs	short-chain fatty acids
TA	title/abstract (screening stage)
TC	total cholesterol
TG	triglycerides
TGR5/GPBAR1	G-protein-coupled bile acid receptor 1
TNF-α	tumor necrosis factor alpha
WAT	white adipose tissue
