# Supplementary material for: Microalgae and Cyanobacteria in the Obesity Evidence Landscape: A PRISMA-ScR Scoping Review with Mechanistic and Safety Mapping
Source: Biology (Basel). 2026 Mar 31;15(7):557. doi: 10.3390/biology15070557 (PMC13072322; doi:10.3390/biology15070557)
Supplement: Supplementary file 1 [file biology-15-00557-s001.zip › Supplementary_File_S4_PRISMA-ScR_Checklist_Version2.pdf]

PRISMA-ScR Checklist (2018) — Microalgae obesity v2

Note: “Page #” can be filled after journal formatting; locations refer to section headings and Supplement IDs (S1–S5).

Section	Item #	Checklist item	Reported in manuscript	Supplement (if any)	Page #
TITLE	1	Identify the report as a scoping review.	Title		p. 1
ABSTRACT	2	Provide a structured summary (background, objectives, eligibility, sources, charting, results, conclusions).	Abstract		p. 1
INTRODUCTION	3	Describe the rationale in the context of existing knowledge.	Introduction		p. 2
INTRODUCTION	4	State the objectives/questions and key elements (concept, context, population).	Objectives (end of Introduction)		p. 2
METHODS	5	Indicate whether a protocol exists/registration (if applicable).	Methods: Design and reporting / Dataset lock and versioning	S1 (search details)	p. 3
METHODS	6	Specify eligibility criteria and rationale.	Methods: Eligibility criteria		p. 4
METHODS	7	Describe information sources, search dates, and interfaces.	Methods: Information sources and search	S1_SearchStrategy	p. 3
METHODS	8	Present the full electronic search strategy for at least one	Methods + Supplement	S1_SearchStrategy	p. 3

		database/sources.			
METHODS	9	Describe selection process (screening/eligibility) and whether it was done in duplicate.	Methods: Selection process	S2_PrimaryHelper (automation semantics, if referenced)	p. 4
METHODS	10	Describe the data charting process (forms, calibration, independent/duplicate).	Methods: Data charting and controlled vocabulary / Automation disclosure	S4_ControlledVocabulary; S5_TableS1_IncludedStudies (charting fields)	p. 4
METHODS	11	List and define all variables sought and any assumptions/simplifications.	Methods: Data charting and controlled vocabulary	S4_ControlledVocabulary; S5_TableS1_IncludedStudies	p. 4
METHODS	12 (optional)	If done, provide rationale for critical appraisal, methods, and how used.	Not done (stated in Methods/Limitations).		p. 3
METHODS	13	Describe methods of handling and summarizing charted data.	Methods: Synthesis and presentation	S5 tables A–D	p. 5
RESULTS	14	Report numbers screened/assessed/included and reasons for exclusions (flow diagram preferred).	Results: Selection of sources + Figure 1	PRISMA_Flow; S3_FTExclusionCodes	p. 6
RESULTS	15	Present characteristics of included sources of evidence with citations.	Results: Study characteristics + Table 1	S5_TableS1_IncludedStudies; S5_Table1_StudyCharacteristics	p. 6
RESULTS	16 (optional)	If done, present results of critical appraisal within sources of evidence.	Not applicable (no critical appraisal performed).		p. 6
RESULTS	17	For each included source, present	Results: Evidence	S5_TableA_Full; S5_TableB_Full;	p. 6

		relevant results that were charted.	maps + Table A–C summaries	S5_TableC_Full	
RESULTS	18	Summarize the charted results in relation to objectives/questions.	Results (Evidence maps + Safety/toxin mapping)	S5 tables + Figure 2–4	p. 6
DISCUSSION	19	Summarize main results, including overview of evidence, concepts, gaps.	Discussion	S5_TableD_MCDA_Rubric (rubric)	p. 24
DISCUSSION	20	Discuss limitations of the scoping review process.	Discussion: Limitations		p. 29
DISCUSSION	21	Provide overall interpretation and potential implications/future directions.	Discussion + Conclusions		pp. 24–29
FUNDING	22	Describe sources of funding for the review and role of funders.	Funding		pp. 42–43
